# 4-Hydr­oxy-1-oxo-1,2-dihydro­phthalazine-6,7-dicarboxylic acid dihydrate

**DOI:** 10.1107/S1600536808014347

**Published:** 2008-06-07

**Authors:** Ling-Ling Liang, Jian-She Zhao, Seik Weng Ng

**Affiliations:** a Department of Chemistry, Shaanxi Key Laboratory for Physico-Inorganic Chemistry, Northwest University, Xi’an 710069, People’s Republic of China; b Department of Chemistry, University of Malaya, 50603 Kuala Lumpur, Malaysia

## Abstract

In the crystal structure of the title compound, C_10_H_6_N_2_O_6_·2H_2_O, the OH and NH groups each serve as a hydrogen-bond donor to one acceptor site whereas the water mol­ecules each serve as a hydrogen-bond donor to two acceptor sites. The hydrogen-bonding scheme gives rise to a three-dimensional network.

## Related literature

For the structure of bis­(hydrazinium) 4-hydr­oxy-1-oxo-*2H*-phthalazine-6,7-dicarboxyl­ate, see: Benniston *et al.* (1999 ▶).
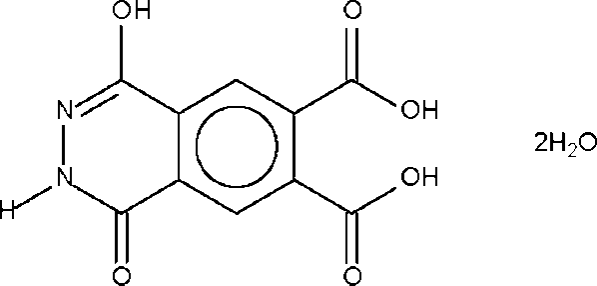


## Experimental

### 

#### Crystal data

C_10_H_6_N_2_O_6_·2H_2_O
*M* *_r_* = 286.20
Triclinic, 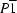

*a* = 6.4069 (1) Å
*b* = 9.4254 (2) Å
*c* = 9.6922 (2) Å
α = 82.843 (2)°
β = 87.496 (1)°
γ = 73.451 (2)°
*V* = 556.65 (2) Å^3^
*Z* = 2
Mo *K*α radiation
μ = 0.15 mm^−1^
*T* = 100 (2) K
0.33 × 0.31 × 0.09 mm

#### Data collection

Bruker SMART APEX diffractometer
Absorption correction: none
4702 measured reflections
2530 independent reflections
2160 reflections with *I* > 2σ(*I*)
*R*_int_ = 0.017

#### Refinement

*R*[*F* ^2^ > 2σ(*F* ^2^)] = 0.035
*wR*(*F* ^2^) = 0.105
*S* = 1.06
2530 reflections
221 parameters
10 restraints
All H-atom parameters refined
Δρ_max_ = 0.43 e Å^−3^
Δρ_min_ = −0.24 e Å^−3^

### 

Data collection: *APEX2* (Bruker, 2007 ▶); cell refinement: *SAINT* (Bruker, 2007 ▶); data reduction: *SAINT*; program(s) used to solve structure: *SHELXS97* (Sheldrick, 2008 ▶); program(s) used to refine structure: *SHELXL97* (Sheldrick, 2008 ▶); molecular graphics: *X-SEED* (Barbour, 2001 ▶); software used to prepare material for publication: *publCIF* (Westrip, 2008 ▶).

## Supplementary Material

Crystal structure: contains datablocks global, I. DOI: 10.1107/S1600536808014347/tk2271sup1.cif

Structure factors: contains datablocks I. DOI: 10.1107/S1600536808014347/tk2271Isup2.hkl

Additional supplementary materials:  crystallographic information; 3D view; checkCIF report

## Figures and Tables

**Table 1:** Hydrogen-bond geometry (Å, °)

*D*—H⋯*A*	*D*—H	H⋯*A*	*D*⋯*A*	*D*—H⋯*A*
O1—H1o⋯O1w	0.84 (1)	1.78 (1)	2.615 (1)	175 (2)
O3—H3o⋯O6^i^	0.84 (1)	1.79 (1)	2.637 (1)	176 (2)
O5—H5o⋯N1^ii^	0.85 (1)	1.91 (1)	2.744 (1)	168 (2)
N2—H2⋯O2w^iii^	0.89 (1)	1.82 (1)	2.695 (1)	167 (2)
O1w—H11⋯O6^iv^	0.85 (1)	1.91 (1)	2.758 (1)	173 (2)
O1w—H12⋯O3^v^	0.85 (1)	2.31 (1)	3.052 (1)	146 (2)
O2w—H21⋯O4	0.84 (1)	1.96 (1)	2.771 (1)	162 (2)
O2w—H22⋯O2^vi^	0.83 (1)	2.37 (2)	3.050 (1)	139 (2)

Symmetry codes: (i) 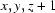; (ii) 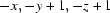; (iii) 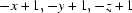; (iv) 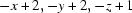; (v) 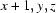; (vi) 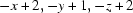.

**Fig. 1.**
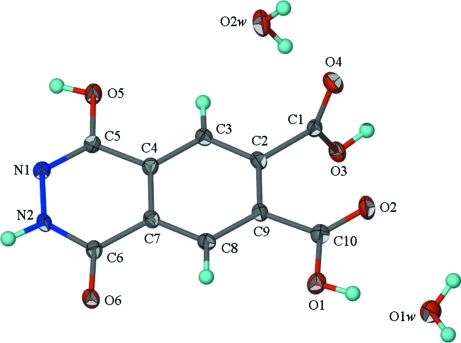
Molecular structure of (I) showing atomic labelling scheme and displacement ellipsoids at the 70% probability level.

## supplementary crystallographic information

### Comment

Benzene-1,2,4,5-tetracarboxylic acid reacts with hydrazine to form bis(hydrazinium) 4-hydroxy-1-oxo-*2H*-phthalazine-6,7-dicarboxylate, whose anion represents a ligand possesses a recognition site for metals as well as a rich hydrogen-bonding motif (Benniston *et al.*, 1999). The neutral acid itself would be more useful for the synthesis of metal derivatives; the neutral acid has been unexpectedly obtained when the reaction was carried out in the presence of a cobaltous salt. The acid crystallizes as a dihydrate (Scheme I, Fig. 1). The –OH and –NH groups each serves as hydrogen-bond donor to one acceptor site whereas the water molecules each serves as hydrogen bond donor to two acceptor sites. The hydrogen bonding scheme gives rise to a three-dimensional network.

### Experimental

Hydrazine hydrate (0.01 g, 0.2 mmol), pyromellitic acid (0.05 g, 0.2 mmol), cobaltous chloride hexahydrate (0.02 g, 0.1 mmol) and water (10 ml) were heated in a 25 ml, Teflon-lined Parr bomb at 433 K for 96 h. The bomb was cooled to room temperature at 10 K per hour.

### Refinement

All hydrogen atoms were located in a difference Fouier map, and were refined with distance restraints (C–H 0.95±0.01, N–H 0.88±0.01 and O–H 0.84±0.01 Å). Temperature factors were freely refined.

### Crystal data

**Table:**

C_10_H_6_N_2_O_6_·2H_2_O	*Z* = 2
*M**_r_* = 286.20	*F*_000_ = 296
Triclinic, *P*1	*D*_x_ = 1.708 Mg m^−^^3^
Hall symbol: -P 1	Mo *K*α radiation λ = 0.71073 Å
*a* = 6.4069 (1) Å	Cell parameters from 2234 reflections
*b* = 9.4254 (2) Å	θ = 2.9–28.2º
*c* = 9.6922 (2) Å	µ = 0.15 mm^−^^1^
α = 82.843 (2)º	*T* = 100 (2) K
β = 87.496 (1)º	Prism, colorless
γ = 73.451 (2)º	0.33 × 0.31 × 0.09 mm
*V* = 556.65 (2) Å^3^	

### Data collection

**Table:**

Bruker SMART APEX diffractometer	2160 reflections with *I* > 2σ(*I*)
Radiation source: fine-focus sealed tube	*R*_int_ = 0.017
Monochromator: graphite	θ_max_ = 27.5º
*T* = 100(2) K	θ_min_ = 2.1º
ω scans	*h* = −8→8
Absorption correction: None	*k* = −12→11
4702 measured reflections	*l* = −12→11
2530 independent reflections	

### Refinement

**Table:**

Refinement on *F*^2^	Secondary atom site location: difference Fourier map
Least-squares matrix: full	Hydrogen site location: inferred from neighbouring sites
*R*[*F*^2^ > 2σ(*F*^2^)] = 0.035	All H-atom parameters refined
*wR*(*F*^2^) = 0.105	*w* = 1/[σ^2^(*F*_o_^2^) + (0.0657*P*)^2^ + 0.0694*P*] where *P* = (*F*_o_^2^ + 2*F*_c_^2^)/3
*S* = 1.06	(Δ/σ)_max_ = 0.001
2530 reflections	Δρ_max_ = 0.43 e Å^−^^3^
221 parameters	Δρ_min_ = −0.24 e Å^−^^3^
10 restraints	Extinction correction: none
Primary atom site location: structure-invariant direct methods	

### Fractional atomic coordinates and isotropic or equivalent isotropic displacement parameters (Å^2^)

**Table:**

	*x*	*y*	*z*	*U*_iso_*/*U*_eq_	
O1	0.92046 (15)	0.93958 (11)	0.64792 (10)	0.0166 (2)	
O2	0.86240 (15)	0.86579 (11)	0.87194 (10)	0.0195 (2)	
O3	0.44292 (15)	0.84970 (10)	1.00234 (9)	0.0155 (2)	
O4	0.68492 (16)	0.62355 (11)	1.02233 (9)	0.0199 (2)	
O5	0.10736 (15)	0.53241 (11)	0.66473 (9)	0.0180 (2)	
O6	0.49784 (14)	0.82489 (10)	0.27322 (9)	0.0147 (2)	
O1W	1.14691 (16)	1.06674 (12)	0.78414 (11)	0.0207 (2)	
O2W	0.85169 (16)	0.35754 (12)	0.90954 (10)	0.0213 (2)	
N1	0.16571 (17)	0.60686 (12)	0.43675 (11)	0.0136 (2)	
N2	0.27394 (17)	0.68218 (12)	0.34120 (11)	0.0134 (2)	
C1	0.5698 (2)	0.73086 (14)	0.95287 (13)	0.0129 (3)	
C2	0.54372 (19)	0.73590 (14)	0.79857 (13)	0.0120 (3)	
C3	0.39766 (19)	0.66628 (14)	0.75724 (13)	0.0126 (3)	
C4	0.35973 (19)	0.67201 (13)	0.61528 (12)	0.0116 (3)	
C5	0.20578 (19)	0.60273 (14)	0.56725 (13)	0.0126 (3)	
C6	0.41699 (19)	0.75457 (14)	0.36872 (13)	0.0117 (3)	
C7	0.46733 (19)	0.74720 (14)	0.51629 (12)	0.0114 (3)	
C8	0.61956 (19)	0.81353 (14)	0.55846 (13)	0.0119 (3)	
C9	0.65794 (19)	0.80760 (14)	0.69923 (12)	0.0117 (3)	
C10	0.8230 (2)	0.87406 (14)	0.74930 (13)	0.0137 (3)	
H10	0.998 (3)	0.980 (2)	0.6878 (19)	0.042 (6)*	
H30	0.458 (3)	0.838 (2)	1.0895 (10)	0.036 (5)*	
H50	0.026 (3)	0.496 (2)	0.622 (2)	0.048 (6)*	
H11	1.253 (2)	1.101 (2)	0.760 (2)	0.044 (6)*	
H12	1.180 (3)	1.0209 (19)	0.8645 (12)	0.034 (5)*	
H21	0.824 (3)	0.4435 (13)	0.9343 (19)	0.034 (5)*	
H22	0.975 (2)	0.314 (3)	0.942 (3)	0.074 (8)*	
H2	0.241 (3)	0.681 (2)	0.2530 (11)	0.037 (5)*	
H3	0.320 (2)	0.6187 (16)	0.8236 (13)	0.017 (4)*	
H8	0.693 (2)	0.8618 (17)	0.4898 (14)	0.019 (4)*	

### Atomic displacement parameters (Å^2^)

**Table:**

	*U*^11^	*U*^22^	*U*^33^	*U*^12^	*U*^13^	*U*^23^
O1	0.0159 (4)	0.0209 (5)	0.0166 (5)	−0.0107 (4)	0.0004 (4)	−0.0025 (4)
O2	0.0220 (5)	0.0270 (6)	0.0137 (5)	−0.0129 (4)	−0.0045 (4)	−0.0019 (4)
O3	0.0206 (5)	0.0168 (5)	0.0094 (4)	−0.0052 (4)	−0.0009 (3)	−0.0032 (4)
O4	0.0267 (5)	0.0175 (5)	0.0140 (5)	−0.0033 (4)	−0.0056 (4)	−0.0007 (4)
O5	0.0223 (5)	0.0245 (5)	0.0128 (5)	−0.0160 (4)	−0.0010 (4)	−0.0007 (4)
O6	0.0173 (4)	0.0192 (5)	0.0096 (4)	−0.0085 (4)	0.0005 (3)	−0.0015 (4)
O1W	0.0195 (5)	0.0238 (6)	0.0217 (5)	−0.0118 (4)	−0.0040 (4)	0.0009 (4)
O2W	0.0232 (5)	0.0215 (6)	0.0187 (5)	−0.0019 (4)	−0.0073 (4)	−0.0075 (4)
N1	0.0151 (5)	0.0144 (5)	0.0128 (5)	−0.0068 (4)	−0.0008 (4)	−0.0009 (4)
N2	0.0175 (5)	0.0161 (6)	0.0083 (5)	−0.0074 (4)	−0.0015 (4)	−0.0013 (4)
C1	0.0152 (6)	0.0155 (6)	0.0108 (6)	−0.0088 (5)	−0.0009 (4)	−0.0011 (5)
C2	0.0128 (6)	0.0120 (6)	0.0102 (6)	−0.0013 (5)	−0.0017 (4)	−0.0016 (5)
C3	0.0143 (6)	0.0139 (6)	0.0101 (6)	−0.0050 (5)	−0.0001 (4)	−0.0007 (5)
C4	0.0122 (6)	0.0104 (6)	0.0120 (6)	−0.0025 (5)	−0.0011 (4)	−0.0025 (5)
C5	0.0143 (6)	0.0115 (6)	0.0123 (6)	−0.0038 (5)	−0.0012 (5)	−0.0017 (5)
C6	0.0120 (5)	0.0123 (6)	0.0108 (6)	−0.0025 (5)	−0.0001 (4)	−0.0031 (4)
C7	0.0119 (5)	0.0113 (6)	0.0102 (6)	−0.0014 (4)	−0.0005 (4)	−0.0023 (5)
C8	0.0125 (6)	0.0120 (6)	0.0107 (6)	−0.0031 (5)	0.0009 (4)	−0.0009 (5)
C9	0.0120 (6)	0.0120 (6)	0.0109 (6)	−0.0026 (5)	−0.0012 (4)	−0.0020 (5)
C10	0.0126 (6)	0.0134 (6)	0.0147 (6)	−0.0025 (5)	−0.0004 (5)	−0.0025 (5)

### Geometric parameters (Å, °)

**Table:**

O1—C10	1.3235 (15)	N2—C6	1.3415 (16)
O1—H10	0.84 (1)	N2—H2	0.89 (1)
O2—C10	1.2143 (16)	C1—C2	1.5054 (17)
O3—C1	1.3139 (15)	C2—C3	1.3838 (17)
O3—H30	0.84 (1)	C2—C9	1.4061 (16)
O4—C1	1.2121 (16)	C3—C4	1.3993 (17)
O5—C5	1.3265 (15)	C3—H3	0.941 (9)
O5—H50	0.85 (1)	C4—C7	1.3961 (16)
O6—C6	1.2511 (15)	C4—C5	1.4487 (16)
O1W—H11	0.85 (1)	C6—C7	1.4688 (17)
O1W—H12	0.85 (1)	C7—C8	1.3983 (17)
O2W—H21	0.84 (1)	C8—C9	1.3884 (17)
O2W—H22	0.83 (1)	C8—H8	0.942 (9)
N1—C5	1.2953 (16)	C9—C10	1.4981 (17)
N1—N2	1.3794 (14)		
			
C10—O1—H10	105.3 (14)	C3—C4—C5	121.13 (11)
C1—O3—H30	107.8 (13)	N1—C5—O5	120.97 (11)
C5—O5—H50	106.0 (15)	N1—C5—C4	122.70 (11)
H11—O1W—H12	104.8 (18)	O5—C5—C4	116.32 (11)
H21—O2W—H22	104 (2)	O6—C6—N2	121.07 (11)
C5—N1—N2	117.77 (10)	O6—C6—C7	123.23 (11)
C6—N2—N1	126.82 (10)	N2—C6—C7	115.70 (11)
C6—N2—H2	119.3 (12)	C4—C7—C8	119.99 (11)
N1—N2—H2	113.9 (12)	C4—C7—C6	118.67 (11)
O4—C1—O3	125.08 (12)	C8—C7—C6	121.35 (11)
O4—C1—C2	122.51 (12)	C9—C8—C7	119.53 (11)
O3—C1—C2	112.24 (10)	C9—C8—H8	121.9 (10)
C3—C2—C9	120.51 (11)	C7—C8—H8	118.6 (10)
C3—C2—C1	116.41 (11)	C8—C9—C2	120.16 (11)
C9—C2—C1	123.07 (11)	C8—C9—C10	121.46 (11)
C2—C3—C4	119.19 (11)	C2—C9—C10	118.37 (11)
C2—C3—H3	120.6 (9)	O2—C10—O1	124.29 (11)
C4—C3—H3	120.2 (10)	O2—C10—C9	122.03 (11)
C7—C4—C3	120.56 (11)	O1—C10—C9	113.68 (11)
C7—C4—C5	118.31 (11)		
			
C5—N1—N2—C6	−0.85 (19)	C3—C4—C7—C6	−177.98 (11)
O4—C1—C2—C3	81.31 (15)	C5—C4—C7—C6	0.97 (17)
O3—C1—C2—C3	−94.09 (13)	O6—C6—C7—C4	176.75 (11)
O4—C1—C2—C9	−99.19 (15)	N2—C6—C7—C4	−2.25 (17)
O3—C1—C2—C9	85.40 (14)	O6—C6—C7—C8	−3.27 (19)
C9—C2—C3—C4	−1.98 (19)	N2—C6—C7—C8	177.73 (11)
C1—C2—C3—C4	177.53 (11)	C4—C7—C8—C9	−1.82 (19)
C2—C3—C4—C7	−0.12 (19)	C6—C7—C8—C9	178.19 (11)
C2—C3—C4—C5	−179.04 (11)	C7—C8—C9—C2	−0.26 (19)
N2—N1—C5—O5	179.93 (10)	C7—C8—C9—C10	178.62 (11)
N2—N1—C5—C4	−0.70 (18)	C3—C2—C9—C8	2.19 (19)
C7—C4—C5—N1	0.56 (19)	C1—C2—C9—C8	−177.29 (11)
C3—C4—C5—N1	179.50 (12)	C3—C2—C9—C10	−176.73 (11)
C7—C4—C5—O5	179.95 (11)	C1—C2—C9—C10	3.80 (18)
C3—C4—C5—O5	−1.10 (18)	C8—C9—C10—O2	−177.84 (12)
N1—N2—C6—O6	−176.72 (11)	C2—C9—C10—O2	1.06 (19)
N1—N2—C6—C7	2.30 (18)	C8—C9—C10—O1	1.46 (17)
C3—C4—C7—C8	2.03 (19)	C2—C9—C10—O1	−179.64 (10)
C5—C4—C7—C8	−179.02 (11)		

### Hydrogen-bond geometry (Å, °)

**Table:**

*D*—H···*A*	*D*—H	H···*A*	*D*···*A*	*D*—H···*A*
O1—H1o···O1w	0.84 (1)	1.78 (1)	2.615 (1)	175 (2)
O3—H3o···O6^i^	0.84 (1)	1.79 (1)	2.637 (1)	176 (2)
O5—H5o···N1^ii^	0.85 (1)	1.91 (1)	2.744 (1)	168 (2)
N2—H2···O2w^iii^	0.89 (1)	1.82 (1)	2.695 (1)	167 (2)
O1w—H11···O6^iv^	0.85 (1)	1.91 (1)	2.758 (1)	173 (2)
O1w—H12···O3^v^	0.85 (1)	2.31 (1)	3.052 (1)	146 (2)
O2w—H21···O4	0.84 (1)	1.96 (1)	2.771 (1)	162 (2)
O2w—H22···O2^vi^	0.83 (1)	2.37 (2)	3.050 (1)	139 (2)

Symmetry codes: (i) *x*, *y*, *z*+1; (ii) −*x*, −*y*+1, −*z*+1; (iii) −*x*+1, −*y*+1, −*z*+1; (iv) −*x*+2, −*y*+2, −*z*+1; (v) *x*+1, *y*, *z*; (vi) −*x*+2, −*y*+1, −*z*+2.
